# Treg cells protect astrocytes from ferroptosis after subarachnoid hemorrhage by activating the HIF-1α/Hmox1 pathway

**DOI:** 10.3389/fimmu.2026.1825459

**Published:** 2026-05-22

**Authors:** Le Huang, Zurong Yao, Fanchao Meng, Yixuan Liu, Yuchen Li, Huaizhang Shi

**Affiliations:** Department of Neurosurgery, The First Affiliated Hospital, Harbin Medical University, Harbin, Heilongjiang, China

**Keywords:** astrocytes, ferroptosis, Hif-1α/Hmox1, regulatory T cells, subarachnoid hemorrhage

## Abstract

**Background:**

Foxp3+ regulatory T (Treg) cells play a central role in maintaining immune homeostasis by suppressing excessive immune responses to prevent autoimmune damage. Within the nervous system, they actively inhibit neuroinflammation and promote tissue repair. Current studies have demonstrated that the number of Tregcells significantly increases following subarachnoid hemorrhage (SAH). Tregcells infiltrating into the brain after SAH can exert critical anti-inflammatory and neuroprotective effects by modulating microglia polarization. Although they are the most abundant and functionally diverse glial cells in the central nervous system, there is only limited research on the direct and specific interactions of astrocytes and Tregcells in SAH. This knowledge gap motivated us to conduct the present studies.

**Methods:**

Establish a mouse model of SAH. Using *in vitro* experiments, Tregcells were co-cultured with astrocytes, using oxygenated hemoglobin (OxyHb) to induce a pathological environment mimicking SAH. Key signaling pathways were identified through transcriptome sequencing. The effects of Tregcells on astrocytes were evaluated through AAV-mediated gene overexpression and knockdown, drug agonists and antagonists, plasmid transfection, SAH grading, neurobehavioral testing, western blotting, and immunofluorescence.

**Results:**

Following SAH, the HIF-1α/Hmox1 signaling axis was activated in the brain, predominantly in astrocytes. However, this compensatory response was insufficient to prevent progressive ferroptotic injury, as indicated by sustained lipid peroxidation and iron accumulation. Tregcells rapidly accumulated after SAH, and enhancement of their function significantly improved neurological outcomes.Transcriptomic profiling of astrocytes revealed that the interaction between Tregcells and astrocyte selectively enriched ferroptosis pathways, with prominent activation of HIF-1α signaling and robust upregulation of Hmox1. Mechanistically, Tregcells promoted a distinct regulatory pattern characterized by moderate stabilization of HIF-1α but disproportionately enhanced Hmox1 expression. Importantly, Tregcells did not induce excessive activation of the HIF-1α/Hmox1 axis, indicating that they maintain this pathway within a defined protective range to suppress ferroptosis without exacerbating toxicity dependent on iron.

**Conclusion:**

This study identified a previously unrecognized immunometabolic mechanism by which Tregcells constrain astrocytic ferroptosis after SAH. By reinforcing an otherwise insufficient endogenous stress response, Tregcells fine tune the HIF-1α/Hmox1 axis to a protective range. They thereby limit oxidative damage that depends on iron and preserve astrocyte integrity.

## 1 Introduction

Subarachnoid hemorrhage (SAH) is a devastating form of stroke associated with high mortality and long-term neurological disability (1, 2). Despite advances in surgical and intensive care management, effective therapies targeting secondary brain injury remain limited (3). Increasing evidence suggests that brain injury after SAH is not solely driven by neuronal loss but involves complex interactions among multiple neural and immune cell populations within the injured microenvironment (4, 5).

Astrocytes, as the most abundant glial cells in the central nervous system, play essential roles in maintaining blood-brain barrier integrity, regulating synaptic transmission, and coordinating inflammatory responses (6, 7). Following SAH, astrocytes undergo profound functional and phenotypic alterations that critically influence neuronal survival and neurological recovery (8). However, the cellular programs that determine astrocyte fate under stress conditions following hemorrhage remain incompletely understood. The same applies to the mechanisms that preserve astrocytic homeostasis during brain injury (9).

Regulatory T cells (Tregcells) have emerged as important modulators of neuroinflammation and tissue repair in various models of acute brain injury, including ischemic stroke and SAH (10, 11). Previous studies have shown that Tregcells can attenuate secondary injury and improve neurological outcomes, primarily by suppressing excessive inflammation and modulating innate immune responses (12). Nevertheless, current research has largely focused on Tregcells interactions with microglia or neurons (13, 14). Whether Tregcells directly influence astrocyte biology after SAH remains largely unexplored. Given the central role of astrocytes in shaping the microenvironment after injury, it is conceivable that Tregcells may exert neuroprotective effects by modulating astrocytic stress responses. This would be an alternative to acting exclusively on classical immune targets. However, the molecular basis underlying potential interactions between Tregcells and astrocytes, and how such interactions contribute to functional recovery after SAH, remain unknown (14, 15).

To address this knowledge gap, we sought to investigate how Tregcells influence astrocyte function under the stress conditions associated with SAH. Following SAH, the functional state of astrocytes directly determines neuronal survival and neurological recovery, and astrocytic injury is considered a critical component of secondary brain injury (16). Meanwhile, Tregcells have been shown in central nervous system disorders not only to modulate immune responses but also to directly affect the activation status and functional phenotype of astrocytes (17). Based on these observations, we propose that Tregcells may exert neuroprotective effects after SAH by modulating astrocytic stress responses.

Ferroptosis is a form of regulated cell death driven by iron dysregulation and lipid peroxidation. These pathological features closely mirror the microenvironment after hemorrhage, raising the possibility that ferroptosis contributes to astrocytic injury in this context. However, whether Tregcells can intervene in this process remains unclear, and the underlying molecular pathways have not been systematically explored. This study aims to experimentally test this hypothesis and provide new insights into the crosstalk mechanisms between immune and glial cells following SAH.

## 2 Materials and methods

### 2.1 Animal and SAH model

This study employed male C57BL/6J mice weighing 20–25g, supplied by Liaoning Changsheng Biotechnology Co., Ltd. The experimental animals were housed in facilities with strictly controlled environmental conditions. These conditions included a temperature of 22-24 °C, relative humidity of 50-60%, and a 12 hour cycle of light and dark (18). Throughout the adaptation period and experimental procedures, the animals were provided free access to standard feed and clean drinking water. The protocol strictly complied with the National Institutes of Health (NIH) guidelines for laboratory animal use and welfare. It was also approved by the Animal Ethics Committee of Harbin Medical University First Affiliated Hospital. The SAH model in mice was constructed using intravascular puncture (19), with procedures adapted from and refined based on prior literature. The steps were as follows: After intraperitoneal injection of sodium pentobarbital (40 mg/kg) for anesthesia (20), the animals were positioned supine and fixed. A midline cervical incision was made to expose the left common carotid artery, external carotid artery, and internal carotid artery. The distal end of the external carotid artery was ligated, followed by a small incision at its proximal end. A 6–0 nylon monofilament was gently introduced into the internal carotid artery, advancing slowly toward the intracranial direction for approximately 12–15 mm until significant resistance was detected. This resistance indicated arrival near the origin of the middle cerebral artery at the Willis circle. A slight thrust was applied to penetrate the vessel wall, maintaining the puncture for about 10 seconds before withdrawing the suture. The sham surgery group received identical anesthesia and vascular exposure procedures without performing puncture (21).

### 2.2 Experimental design

**Figure 1:**
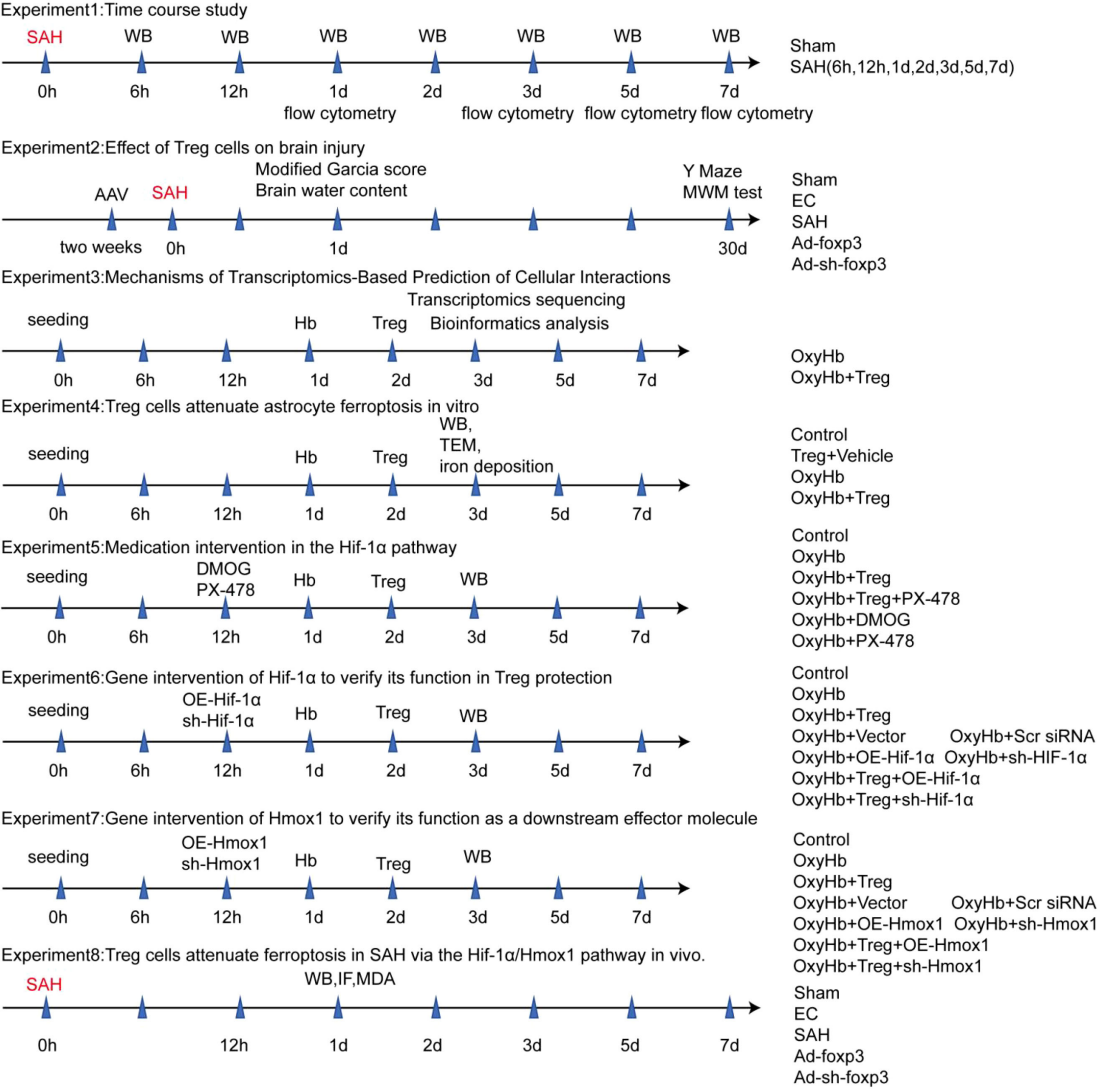
Schematic diagram of the experimental design.

The schematic diagram of the experimental design is shown in **Figure 1**. The experiments were organized into two main categories. Experiments 1, 2, and 8 were conducted *in vivo* using the mouse SAH model, while Experiments 3–7 were performed *in vitro* using astrocyte cultures. Within each category, the experiments were further divided according to the specific study objectives as described below.

#### 2.2.1 Experiment 1

The first experiment was designed to characterize the temporal profile of Tregcells infiltration in the brain following SAH. A mouse model of SAH was established, and brain tissues were collected at predefined time points (days 1, 3, 5, and 7) after injury to assess dynamic changes in Tregcells abundance within the central nervous system.Time points for Tregcells analysis were selected based on the known time course of secondary brain injury after SAH, with pilot experiments confirming reliable detection of Tregcells at each interval. Day 1 was chosen to capture early infiltration events, while days 3, 5, and 7 were selected to evaluate sustained accumulation during the acute and subacute phases.In parallel, protein expression of Foxp3 in brain regions adjacent to the hemorrhage was examined by western blotting. This provided independent validation of Tregcells accumulation at the tissue level. Immunofluorescence staining was further performed to localize cells positive for Foxp3 within brain sections and to assess their spatial distribution following SAH.

#### 2.2.2 Experiment 2

For therapeutic manipulation of Tregcells, mice received a single intracerebral injection (2 μL) of adeno-associated virus (AAV) two weeks prior to SAH model establishment. The AAV vectors used were overexpressing Foxp3 (Ad-Foxp3), knockdown of Foxp3 (Ad-sh-Foxp3), or empty control (EC). Neurological function scores, behavioral tests, and brain water content measurements were performed 24 h after SAH establishment (n = 12 per group).

#### 2.2.3 Experiment 3

Under different conditions, astrocytes were co-cultured with Tregcells, and RNA was extracted from the astrocytes using Trizol lysis method to prepare batch RNA sequencing libraries. After quality control, downstream analyses were performed using R packages such as edgeR and GOplot for data visualization.

#### 2.2.4 Experiment 4

The key components of the hypoxia stress signaling pathway were detected by western blotting. This was done to investigate the potential regulatory mechanisms underlying the astrocyte stress response mediated by Tregcells. The expression levels of core ferroptosis proteins (including GPX4, SLC7A11 and COX2) were assessed by western blotting to evaluate changes in antioxidant defense mechanisms and lipid peroxidation status. Meanwhile, the functional status of redox homeostasis and antioxidant systems that depend on glutathione was evaluated by quantifying intracellular glutathione (GSH) levels. Subsequently, transmission electron microscopy (TEM) was employed to examine the ultrastructure of mitochondria in astrocytes.

#### 2.2.5 Experiment 5

To regulate the HIF-1α signaling pathway, the stabilizer DMOG and inhibitor PX-478 were used, respectively. After drug treatment, the protein expression levels of HIF-1α and its downstream target Hmox1 were detected by western blotting to validate pathway regulation. Simultaneously, key ferroptosis proteins were examined to assess antioxidant capacity and downstream changes in processes related to lipid peroxidation.

#### 2.2.6 Experiment 6

Astrocytes were transfected with plasmids to enhance or inhibit HIF-1α expression, with corresponding control plasmids set to exclude transfection effects. After transfection, the astrocytes were exposed to oxyhemoglobin (OxyHb) to simulate the microenvironment following SAH. The protein expression levels of HIF-1α were detected by western blotting to validate the efficiency of gene manipulation. Concurrently, the expression levels of Hmox1 were measured as a representative downstream effector of the HIF-1α signaling pathway. To assess the impact of HIF-1α regulation on ferroptosis processes, key ferroptosis proteins, including GPX4, SLC7A11, and COX2, were analyzed.

#### 2.2.7 Experiment 7

Hmox1 overexpression in astrocytes was achieved by transfecting a plasmid encoding Hmox1. Endogenous Hmox1 expression was suppressed by transfecting astrocytes with a short hairpin RNA (shRNA) construct targeting Hmox1. Corresponding control plasmids were included in the experiment to exclude transfection effects. After gene manipulation, the astrocytes were exposed to OxyHb to simulate the microenvironment following SAH. The changes in Hmox1 expression after transfection were detected by western blotting. To evaluate the feedback effects of the upstream signaling pathway, the levels of HIF-1α protein were also measured.

#### 2.2.8 Experiment 8

Consistent with Experiment 2, animals were grouped according to Tregcells manipulation regulated by Foxp3. After SAH induction, protein blot analysis was performed on brain tissue samples to detect the expression levels of ferroptosis key proteins. Additionally, lipid peroxidation was quantitatively analyzed by measuring malondialdehyde (MDA) levels. Immunofluorescence staining was employed to observe and detect spatial localization of ferroptosis markers such as GPX4, HIF-1α, and Hmox1. Finally, Prussian blue staining was used to assess intracerebral iron deposition.

### 2.3 Drug administration

For *in vivo* modulation of Tregcells, recombinant AAV vectors carrying overexpression or knockdown of Foxp3 constructs were stereotaxically injected into the lateral ventricles of mice. Each mouse received 2 μL of AAV two weeks prior to SAH induction. For *in vitro* experiments, DMOG and PX-478 were directly added to the culture medium to modulate HIF-1α activity. DMOG and PX-478 were used to stabilize or inhibit HIF-1α activity, respectively. The concentrations were determined based on preliminary dose-response experiments in OxyHb-stimulated astrocytes. DMOG was used at 500μM and PX-478 at 50μM. These concentrations reproducibly modulated HIF-1α expression without inducing significant cytotoxicity, and are consistent with previous studies using similar experimental systems (22, 23).In addition, transfection using plasmids was used in Experiments 6 and 7 to genetically manipulate HIF-1α or Hmox1 expression in astrocytes.Plasmids encoding HIF-1α or Hmox1 overexpression, as well as corresponding shRNA constructs, were introduced into cells using standard transfection protocols. Appropriate vehicle and vector controls were included in all experiments.

### 2.4 SAH grade

The success and severity of the SAH model were independently assessed by two researchers using a blinded method. At 24 h after model creation, mice were deeply anesthetized and perfusionally euthanized to completely remove brain tissue. The ventral side of the brain (basal cistern) was then divided into six adjacent regions based on vascular distribution characteristics. Each region’s hemorrhage severity was scored according to established criteria, with scores ranging from 0 (no visible hemorrhage) to 18 (extensive clot coverage obscuring the Willis circle structure) (24). The total SAH score for each animal was calculated by summing scores from all six regions. To ensure model consistency, only mice scoring ≥8 points were included in subsequent experimental analysis, while those below this threshold were excluded.

### 2.5 Short-term neurological function evaluation

Neurological function assessment was conducted 24 h after SAH modeling by two unblinded researchers. Short-term neurological evaluation utilized the modified Garcia scoring scale combined with the balance beam test. The modified Garcia scoring system included six behavioral dimensions: 1) voluntary movement; 2) limb motor coordination; 3) bilateral forelimb extension ability; 4) grid climbing ability; 5) somatosensory perception; 6) whisker touch response. Each item was scored on a 0–3 or 1–3 scale based on neurological deficits, with total scores ranging from 3 (severe neurological impairment) to 18 (normal function). The balance beam test assessed motor coordination and balance by placing mice on a circular beam of 1 cm diameter, recording their performance over 60 s. Scoring criteria included walking stability and fall frequency, with scores ranging from 0 (unable to remain on the beam) to 4 (steady walking without falls). Each mouse underwent three consecutive tests, and the median score was used as the final neurological function score to minimize behavioral variability errors.

### 2.6 Long-term neurological function evaluation

The Y-maze was used to assess spontaneous alternation behavior in mice, reflecting their spatial working memory capacity (25). The experimental setup consisted of three arms of equal length (labeled A, B, and C) arranged at angles of 120 degrees. During testing, mice were gently placed at the end of any arm and allowed to freely explore the maze for 8 min. The entire exploration process was automatically recorded by video tracking systems mounted on the ceiling. By analyzing the recorded sequences, the following core metrics were calculated: spontaneous alternation accuracy (the percentage of consecutive entries into three different arms relative to total possible alternations).

Spatial learning and memory were evaluated using the Morris water maze. Mice were trained to locate a submerged escape platform in a circular pool filled with opaque water (22 ± 1 °C). Training consisted of four trials per day for six consecutive days, and escape latency was recorded. A probe trial was conducted 24 h after the final training session with the platform removed. Spatial memory was assessed by the time spent in the target quadrant and the number of crossings over the former platform location (26). Swimming speed was measured to exclude motor deficits. Behavioral tracking and analysis were performed using an automated video tracking system, with investigators blinded to group allocation.

### 2.7 Brain edema

The evaluation of cerebral edema employed the classic wet and dry weight method. The mice were euthanatized 24 h after SAH modeling and their intact brain tissues were rapidly extracted. The brain was placed on an ice disk for separation, surface fluid was gently aspirated with filter paper, and the brain was immediately weighed to record the wet weight. Subsequently, the brain tissue was placed in an incubator at 105 °C for 72 consecutive until weight stabilization. The measured mass then designated as dry weight. Brain tissue water content was calculated using the formula: [(wet weight-dry weight)/wet weight] × 100%, which directly reflects the severity of cerebral edema (27).

### 2.8 Western blot analysis

Total protein was isolated from brain tissue samples or cultured astrocytes with ice cold RIPA lysis buffer (Beyotime Biotechnology, China). The buffer fortified with a complete protease and phosphatase inhibitor cocktail to preserve protein integrity and phosphorylation states. Protein concentration was determined via a standard BCA assay. Subsequently, equivalent amounts of protein lysates were subjected to electrophoretic separation on 10-12.5% sodium dodecyl sulfate polyacrylamide gels (SDS-PAGE). They were then transferred onto activated PVDF membranes. To prevent nonspecific antibody binding, the membranes were incubated in a blocking solution of 5% nonfat milk. Immunoblotting was performed by incubating the membranes with specific primary antibodies targeting Hmox1, HIF-1α, SLC7A11, GPX4, Foxp3 and β-actin (all from Proteintech) at 4 °C overnight. Following extensive washes, the membranes were exposed to appropriate secondary antibodies for 1 h at room temperature. Signal detection was achieved using an enhanced chemiluminescence substrate (NCM Biotechnology, China), and the resulting band intensities were quantified densitometrically using ImageJ software. For each sample, the target protein expression level was normalized to its corresponding β-actin signal.

### 2.9 Immunofluorescence staining

Immunofluorescence analysis was conducted to assess protein expression and cellular localization in fixed brain sections (28). Immunofluorescence analysis was performed on coronal brain sections encompassing the peri-hemorrhagic region. Specifically, sections were taken at the level of the dorsal hippocampus and the overlying cerebral cortex, where subarachnoid hemorrhage was most prominent. These regions were selected because they are consistently affected in this SAH model and are functionally linked to the sensorimotor and cognitive outcomes assessed in the study.Briefly, sections were fixed in 4% paraformaldehyde. To minimize nonspecific binding, sections were blocked and permeabilized for 1 h at room temperature. The solution used was 5% BSA and 0.1% Triton X-100 in PBS. After thorough rinsing with PBS, the sections were probed with specific primary antibodies (Proteintech) diluted at 1:500 in blocking buffer. These included rabbit anti-Foxp3, rabbit anti-Hmox1, mouse anti-HIF-1α, and mouse anti-GPX4, and incubated overnight at 4 °C. Following three 5 min washes with PBS to remove unbound primary antibodies, the sections were incubated with fluorescent secondary antibodies appropriate for the species. The incubation was performed for 1 h at room temperature. The secondary antibodies were diluted 1:1000, and the incubation was shielded from light. Images were acquired using a fluorescence microscope.

### 2.10 Assessment of MDA and GSH

Cortical tissues from mice were homogenized, and the resulting homogenates were centrifuged to collect the supernatant for subsequent biochemical analyses. According to the manufacturers’ instructions, the levels of MDA were determined using specific commercial assay kits (Beyotime Biotechnology, China). In astrocytes, intracellular glutathione GSH levels were detected after co-culture with either OxyHb or Tregcells. Cells were collected after treatment, and total GSH content was determined using a GSH assay kit. To account for differences in cell density, the results were normalized by protein concentration.

### 2.11 TEM

To evaluate mitochondrial ultrastructure, astrocyte samples from all groups were processed for TEM (29). The cells were first fixed at 4 °C with 2.5% glutaraldehyde for 4 h, then dehydrated and embedded in resin. Using an ultrathin sectioner, the aggregates were cut into 60 nm ultrathin sections(Leica, Germany). Subsequently, the sections were observed under a Zeiss transmission electron microscope (USA), and highly resolved images were systematically acquired to assess mitochondrial morphology.

### 2.12 Flow cytometry

Suspensions of single cells were prepared from the left cerebral hemisphere. This allowed us to characterize the immune cell populations that infiltrate the brain, especially Tregcells, by flow cytometry. Briefly, mice were deeply anesthetized with pentobarbital and subjected to transcardial perfusion with PBS to remove circulating blood cells. The collected brain tissue was mechanically ground and subjected to enzymatic digestion, ultimately yielding a suspension of single cells. The procedures were strictly performed in accordance with the experimental protocols provided by the manufacturer. The homogenate was filtered through a 70 μm nylon filter to remove debris, followed by treatment with 1X erythrocyte lysis buffer to eliminate red blood cells. Subsequently, the sample was stained with a set of surface marker antibodies at 4 °C. Foxp3 is a key transcription factor defining regulatory T cells. To detect its intracellular expression, the cells were fixed and permeabilized using a Foxp3 transcription factor staining kit. This was followed by incubation with anti-Foxp3 antibodies. All samples were collected via flow cytometry, and the data were analyzed using FlowJo.

### 2.13 Bulk RNA sequencing and analysis

To investigate the transcriptional changes in astrocytes induced by Tregcells, we performed bulk RNA sequencing. Tregcells were bought from Shanghai Cell Bank. Viability was checked by trypan blue staining. Cells were rested for 2 hours before co-culture.Astrocytes were purified from the co-culture system, and total RNA was isolated using Trizol reagent. Sequencing libraries were constructed and sequenced on an Illumina platform, generating reads with paired ends. The raw sequencing data underwent stringent quality control using FastQC and were aligned to the reference genome. Differential gene expression analysis between experimental groups was conducted using the edgeR package in R. The significance threshold set at |log2(fold change)| > 1 and an adjusted P value < 0.05. To interpret the biological significance of the altered transcriptome, Gene Ontology (GO) enrichment analysis was performed. The results were visualized using the GOplot package to create insightful circular plots and other graphics, elucidating the functional pathways modulated by Tregcells interactions.

### 2.14 Statistical analysis

All quantitative data in this study are presented as mean ± standard deviation (SD). For comparisons between groups, we employed appropriate statistical methods based on data characteristics and experimental design. Independent samples t test was used for comparing two groups with normal distribution. One-way ANOVA was applied for differences among multiple groups. For neurofunctional data measured at multiple time points, two-way repeated measures ANOVA was used for evaluation. All statistical analyses and figure generation were performed using GraphPad Prism software (version 9.0.0, GraphPad Software Inc., USA). The statistical significance threshold was set at P < 0.05.

## 3 Results

### 3.1 Pathological changes in the brain after SAH, Tregcells infiltration and upregulation of Foxp3 expression

**Figure 2:**
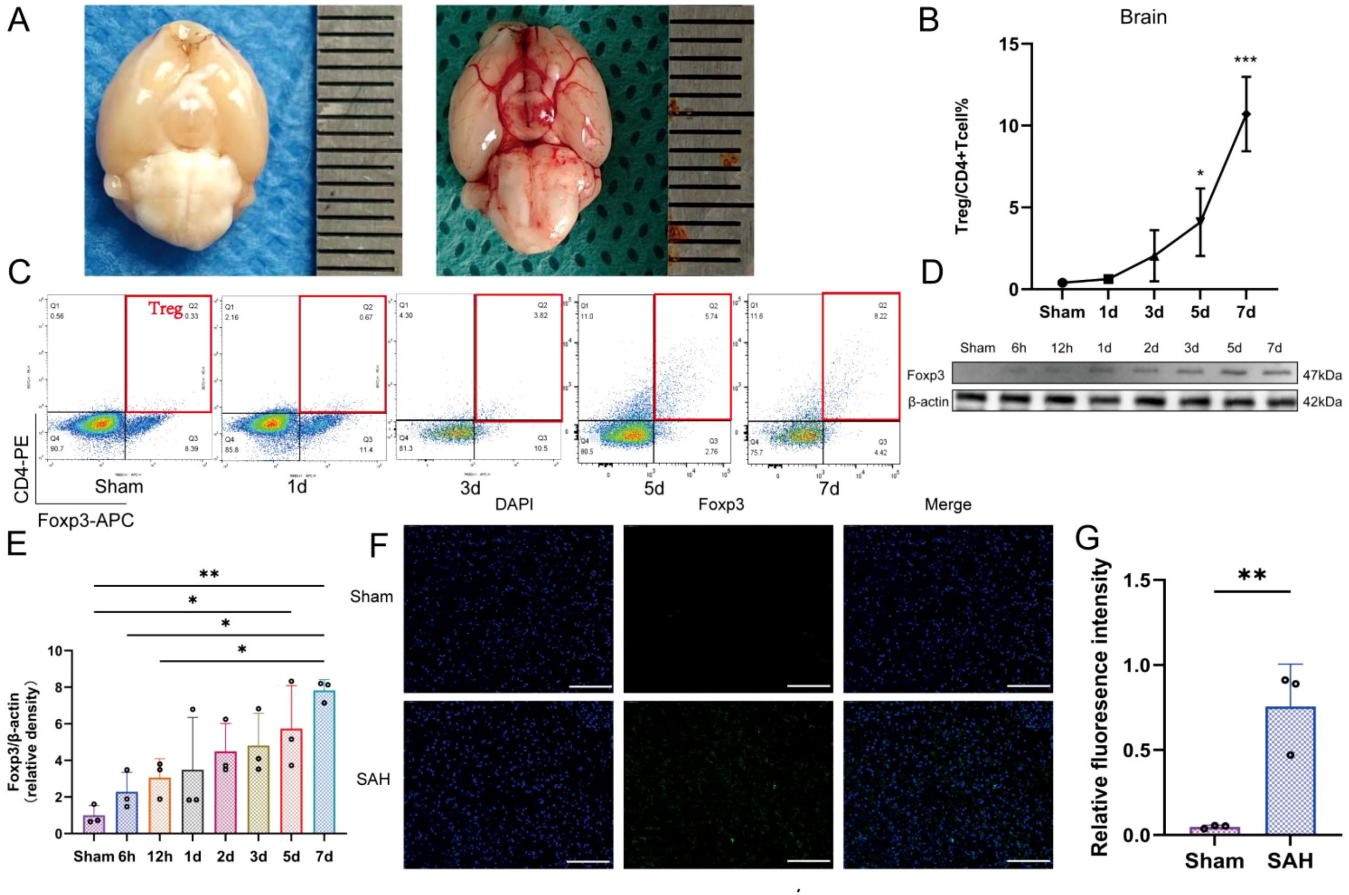
SAH induces early accumulation of Tregcells in the brain. **(A)** Representative brain images from Sham and SAH mice. **(B)** Quantification of brain infiltrating Tregcells at 1, 3, 5, and 7 days after SAH, n = 6. **(C)** Representative flow cytometry plots of CD4^+^CD25^+^Foxp3^+^ Tregcells. **(D)** Western blot analysis of Foxp3 expression at indicated time points. **(E)** Quantification of Foxp3 protein levels normalized to β-actin, n = 3. **(F)** Representative immunofluorescence images of Foxp3 in Sham and SAH groups. **(G)** Quantification of cells positive for Foxp3 from immunofluorescence analysis, n = 3. *P < 0.05, **P < 0.01, ***P < 0.001.

To evaluate the SAH model and investigate its early immune cell response, we first examined brain tissue morphology and analyzed the dynamic changes in Tregcells. The intact brain tissue of sham mice was examined. A distinct hemorrhagic mass was observed in the basal pool of the brain 24 h after SAH modeling. This confirming the successful establishment of the model (**Figure 2A**).

Subsequently, we performed quantitative analysis using flow cytometry to assess the infiltration of Tregcells in the brain at different time points (1, 3, 5 and 7 days) following SAH. Tregcells were defined as CD4^+^CD25^+^Foxp3^+^ cells. Representative flow cytometry plots demonstrated an increased proportion of CD4^+^CD25^+^Foxp3^+^ cells in the brain after SAH (**Figure 2B**). Quantitative analysis further revealed that Tregcells exhibited significant infiltration as early as day 1 after SAH. They maintained high levels at days 5 and 7 (**Figure 2C**).

To validate this change at the molecular level, we measured the expression of Foxp3, a key transcription factor in Tregcells. Western blot results showed a temporal upregulation of Foxp3 protein expression in the brain tissue following SAH. The upregulation was significant (**Figures 2D, E**). Immunofluorescence staining further confirmed the upregulation of Foxp3 expression in the SAH group compared to the sham group (**Figure 2F**, P < 0.01). These findings collectively demonstrate that SAH not only induces significant pathological changes in brain tissue. It also rapidly triggers and sustains the infiltration and activation of Tregcells in the brain. The core regulatory factor Foxp3 is upregulated.

### 3.2 The protective effect of Tregcells on neurological outcomes after SAH by regulating Foxp3

**Figure 3:**
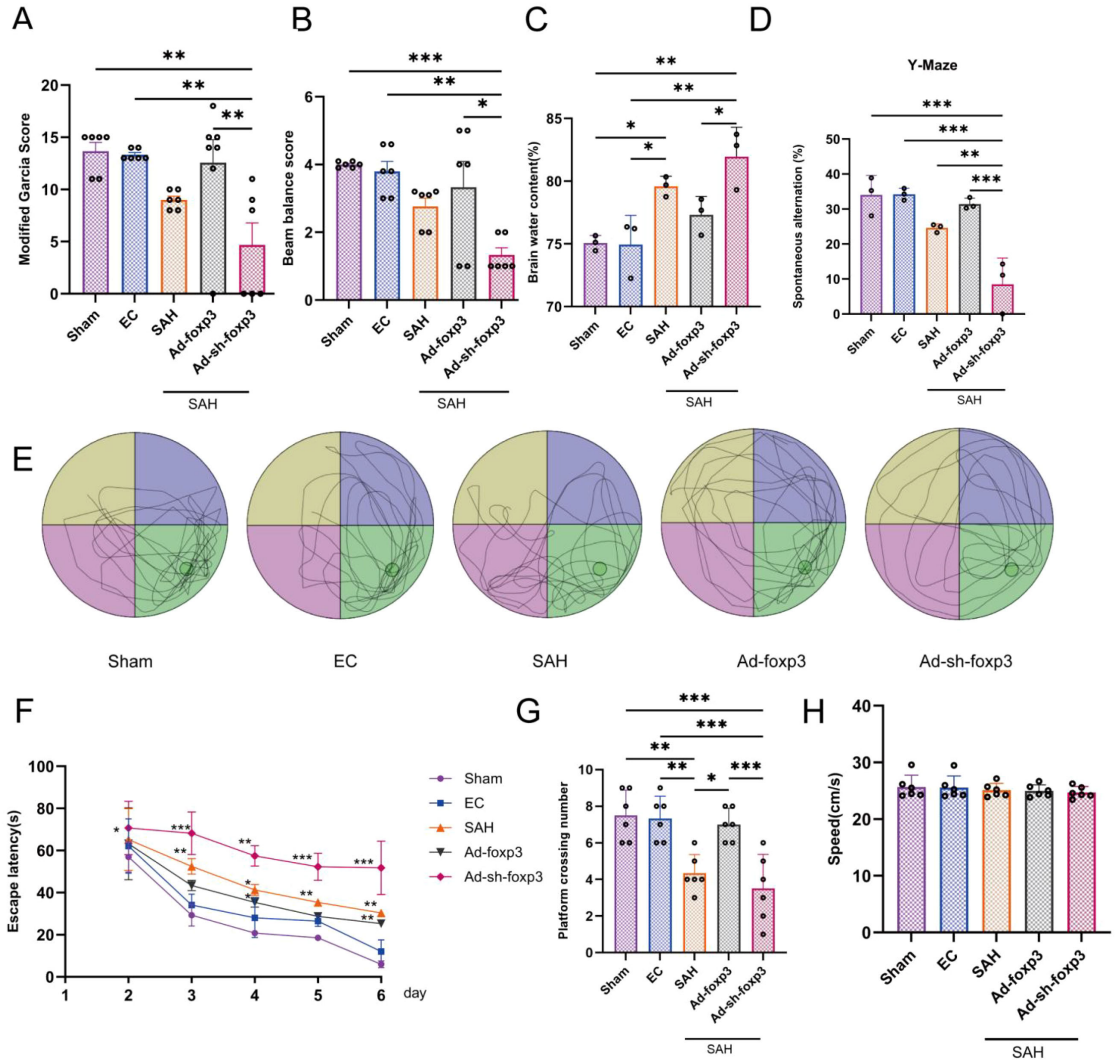
Tregcells regulation dependent on Foxp3 improves neurological and cognitive outcomes after SAH. **(A)** Modified Garcia neurological scores. **(B)** Balance beam test performance. **(C)** Brain water content. **(D)** Y-maze spontaneous alternation rate. **(E)** Morris water maze escape latency. **(F)** Representative swimming trajectories in the Morris water maze. **(G)** Number of crossings over the platform. **(H)** Swimming speed. n = 6 per group. *P < 0.05, **P < 0.01, ***P < 0.001.

Having established the infiltration of Tregcells after SAH, we sought to define their functional significance. We found that Foxp3, the master transcription factor of Tregcells, is a critical regulator of neurological outcomes. In the acute phase, AAV-mediated Foxp3 overexpression conferred robust protection. It significantly alleviated neurological deficits induced by SAH on the modified Garcia scale and beam walking test (**Figures 3A, B**). Concordantly, Foxp3 overexpression markedly reduced brain water content. This demonstrated its efficacy in mitigating cerebral edema (**Figure 3C**). Conversely, Foxp3 knockdown exacerbated both functional impairment and edema. Importantly, this protective effect translated to long-term cognitive recovery. Mice overexpressing Foxp3 displayed significant enhancements in spatial working memory and learning at 21 days after SAH. These were measured by the Y-maze and Morris water maze tests (**Figures 3D, E**). The Morris water maze test began on day 21 after SAH and consisted of six consecutive days of training. During training, escape latency was recorded daily to assess spatial learning (**Figure 3F**). Representative swimming trajectories were recorded on the seventh day (i.e., day 27 post-SAH) (**Figure 3E**), and a probe trial was conducted to evaluate spatial memory by measuring the number of platform crossings (**Figure 3G**). The Y-maze test was performed separately on day 21 after SAH to assess spontaneous alternation behavior (**Figure 3D**).Taken together, these data unequivocally demonstrate that Tregcells, via Foxp3, not only protect against acute neurological injury and edema but also promote lasting cognitive recovery after SAH.

### 3.3 Transcriptomic analysis reveals that Tregcells regulates the gene expression profile of astrocytes and specifically enriches ferroptosis and HIF-1α pathway

**Figure 4:**
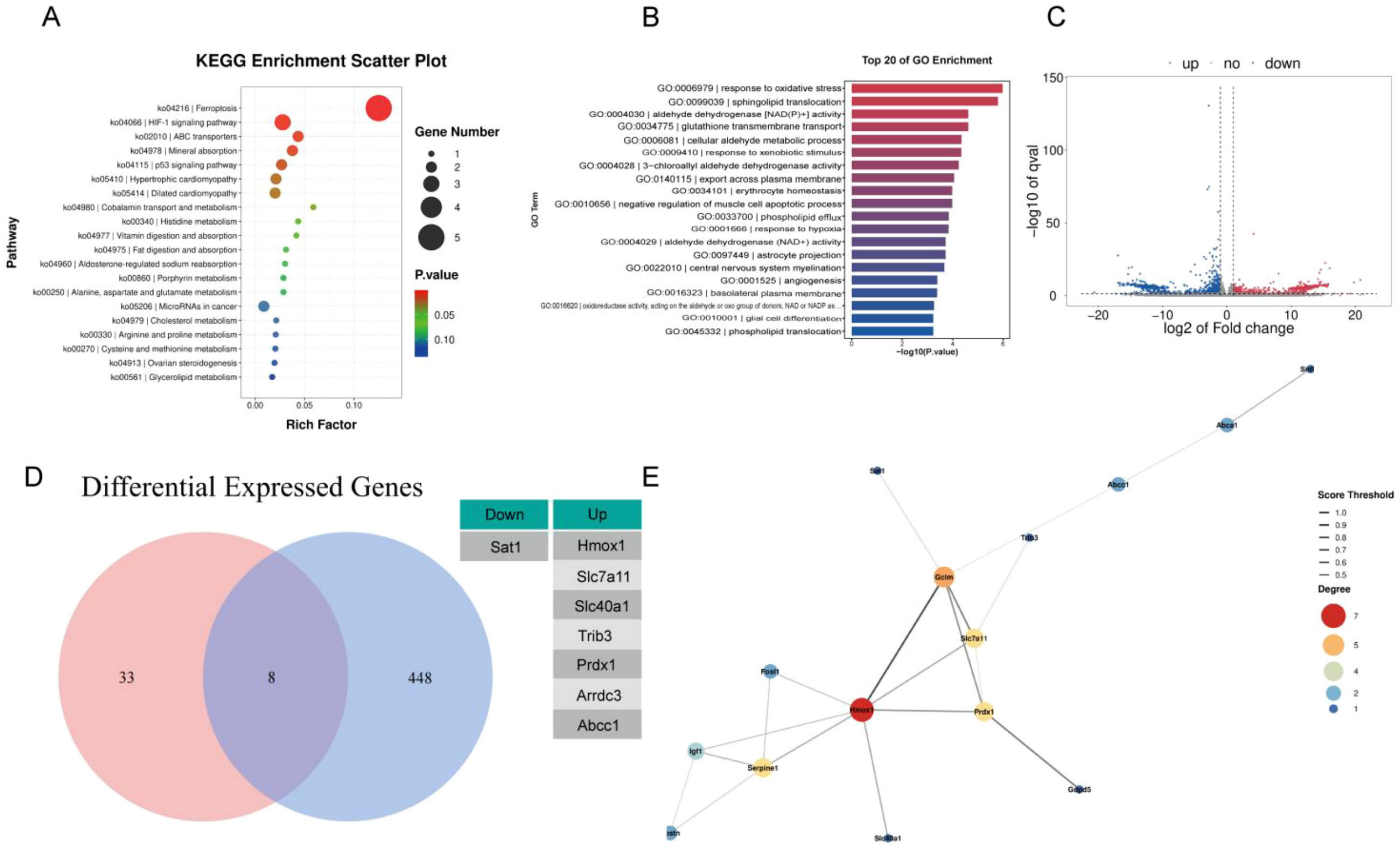
Transcriptomic analysis identifies ferroptosis pathways regulated by Tregcells co-culture. **(A)** KEGG pathway enrichment of differentially expressed genes. **(B)** GO enrichment analysis. **(C)** Volcano plot of differentially expressed genes. **(D)** Overlap between differentially expressed genes and ferroptosis genes. **(E)** Protein-protein interaction network and hub gene analysis. FDR < 0.05.

To systematically investigate the molecular mechanisms by which Tregcells protect astrocytes at the transcriptomic level, we performed RNA-seq analysis on two groups of astrocytes. One group was treated with OxyHb (simulating SAH pathology), and the other was co-cultured with Tregcells after OxyHb treatment (simulating Tregcells intervention). We performed differential expression analysis of the two sample groups using DESeq2 software. The threshold was set at |log_2_FoldChange| ≥ 1 and false discovery rate (FDR) < 0.05. A large number of differentially expressed genes were identified.To elucidate the biological functions of differentially expressed genes, we performed Kyoto Encyclopedia of Genes and Genomes (KEGG) pathway enrichment analysis (hypergeometric test, FDR < 0.05). The results showed that the ferroptosis pathway exhibited the highest significance among all enriched pathways (**Figure 4A**). The plot further confirming the significant enrichment of both this pathway and the HIF-1 signaling pathway.

To characterize the biological functions associated with these differentially expressed genes, Gene Ontology (GO) enrichment analysis was performed. The enriched GO terms spanned biological processes, cellular components, and molecular functions. Prominent enrichment observed in processes related to oxidative stress response, hypoxic response, lipid transport, and glutathione metabolism. Notably, GO terms such as response to oxidative stress, response to hypoxia, glutathione transmembrane transport, and cellular aldehyde metabolic process were among the most significantly enriched biological processes (**Figure 4B**). This indicated substantial remodeling of pathways related to redox in astrocytes following Tregcells.

Consistent with these findings, molecular function analysis revealed enrichment of oxidoreductase activities, particularly those acting on aldehyde or oxo groups with NAD(P)+ as cofactors. Meanwhile, cellular component analysis highlighted structures associated with membranes involved in lipid and phospholipid transport. Several GO terms related to glial cells, including astrocyte projection and glial cell differentiation, were also enriched. This suggests that Tregcells intervention may influence astrocytic functional states beyond inflammatory signaling.

The volcano plot visually demonstrated the overall trend of gene expression changes, with Hmox1 identified as one of the most significantly upregulated genes (**Figure 4C**). We compared the differentially expressed gene set in this study with the ferroptosis gene database (FerrDB). Eight genes were commonly differentially expressed, suggesting that Tregcell intervention specifically affects the ferroptosis molecular programs in astrocytes (**Figure 4D**).

Furthermore, to further identify core molecules in the regulatory network, we constructed a protein interaction network of differentially expressed genes. Hub genes were then screened using multiple topological algorithms. The analysis revealed that multiple genes associated with oxidative stress and iron metabolism were located at the core of the network (**Figure 4E**).

In conclusion, transcriptomic analysis has demonstrated through multidimensional approaches that co-culture with Tregcells can extensively reshape the gene expression profile of astrocytes stimulated by OxyHb. This reshaping included specific and significant enrichment in the HIF-1α signaling pathway and ferroptosis pathway. This provides robust omics evidence supporting the hypothesis that Tregcells inhibit astrocyte ferroptosis by regulating this central pathway.

### 3.4 Tregcells remodels astrocyte stress response and reverses ferroptosis induced by OxyHb

**Figure 5:**
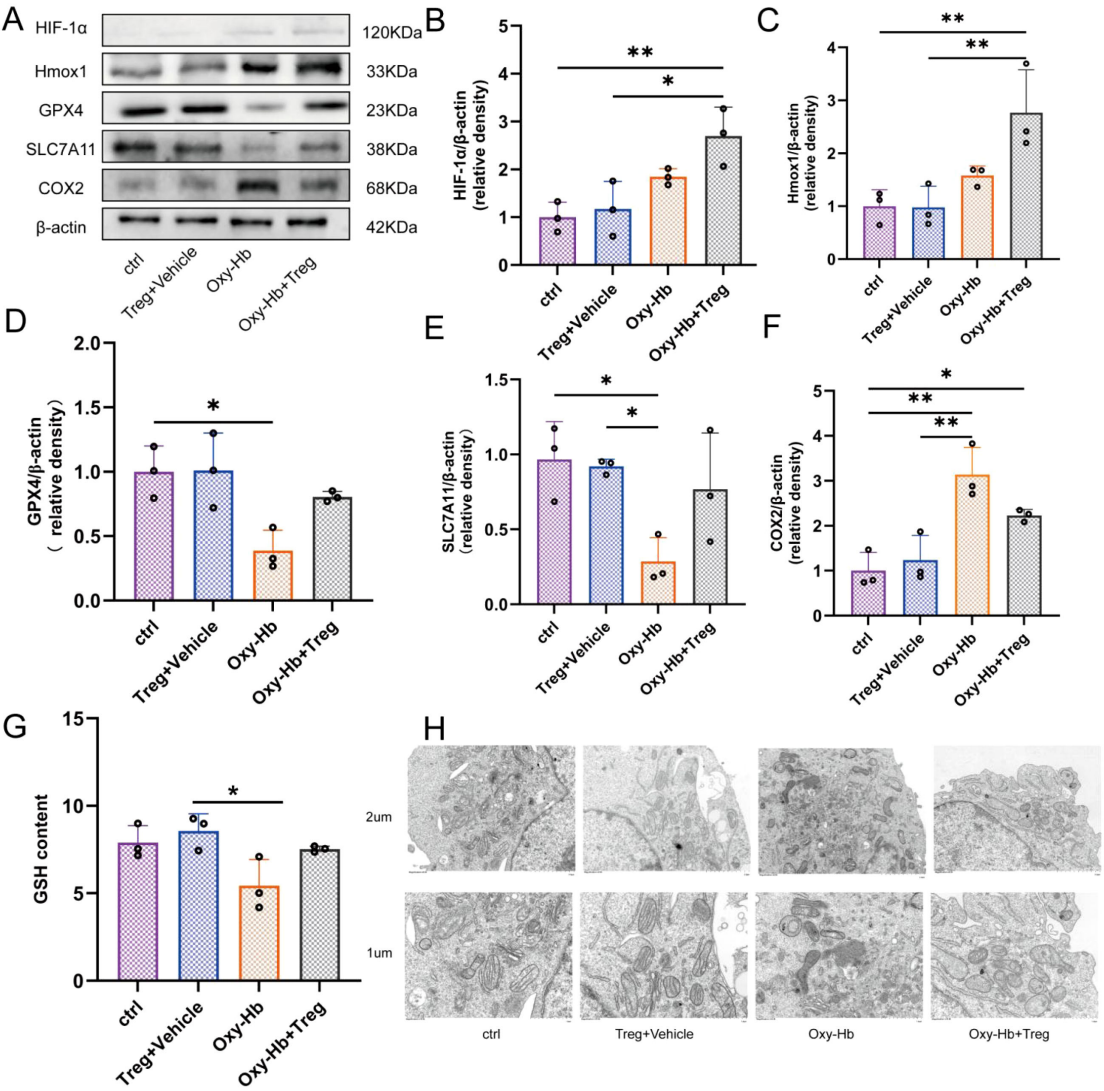
Tregcells co-culture attenuates ferroptosis protein alterations induced by OxyHb in astrocytes. **(A)** Western blot analysis of GPX4, SLC7A11, COX2, HIF-1α, and Hmox1. **(B–F)** Quantification of protein levels normalized to β-actin, n = 3. **(G)** Intracellular glutathione (GSH) content assay; **(H)** TEM observation of mitochondrial ultrastructure. *P < 0.05, **P < 0.01.

To investigate the effect of Tregcells on iron death of astrocytes after SAH, we established an *in vitro* co-culture system and analyzed the results by western blot and TEM. Compared with the control group, no significant changes were observed in protein expression levels in the Tregcells group (**Figure 5A**). After stimulation with OxyHb, astrocytes exhibited a typical ferroptosis molecular phenotype: significant downregulation of key ferroptotic inhibitors GPX4 and SLC7A11, while upregulation of ferroptotic factor COX2. (**Figures 5D–F**). Meanwhile, the expression of the stress protein HIF-1α and its downstream target Hmox1 was upregulated (**Figures 5B, C**), indicating that the cells were in a state of intense hypoxia and oxidative stress.

However, when astrocytes stimulated with OxyHb were co-cultured with Tregcells, the protein expression profile exhibited characteristic alterations. Compared to the OxyHb group, the protein levels of GPX4, SLC7A11, and COX2 were significantly restored (**Figures 5D–F**). Notably, although HIF-1α expression remained higher than that in the OxyHb group, its upregulation was not statistically significant (**Figure 5B**), whereas the protein level of Hmox1 was further significantly elevated (**Figure 5C**).

TEM analysis confirmed the protective effect of Tregcells at the ultrastructural level.The astrocyte mitochondria in the control group and the group with Tregcells and Vehicle exhibited intact morphology (**Figure 5H**). In the OxyHb treatment group, the mitochondria displayed typical ferroptosis characteristics: reduced volume, increased membrane density, and decreased crystalline structure (**Figure 5H**). In contrast, the mitochondrial damage morphology was significantly improved in the OxyHb and Tregcells group (**Figure 5H**). Furthermore, the detection of cellular redox status revealed that OxyHb stimulation significantly reduced the levels of reduced glutathione (GSH) in astrocytes, while co-culture with Tregcells partially reversed the depletion of GSH (**Figure 5G**).

In conclusion, the *in vitro* experimental results indicate that Tregcells may modulate astrocyte stress responses through certain signaling pathways. This transition moves them from an inefficient state that consumes energy to an efficient and precise protective state. This is characterized by a moderate and protective upregulation of Hmox1, ultimately reversing abnormal expression of ferroptosis proteins, mitochondrial damage, and redox imbalance.

### 3.5 Excessive or insufficient HIF-1α activation exacerbates ferroptosis

To further clarify the role of HIF-1α signaling in Tregcells mediated regulation of ferroptosis, pharmacological modulation of HIF-1α was performed in astrocytes stimulated with OxyHb. This modulation used the agonist DMOG and the inhibitor PX-478. All interventions were conducted on the background of OxyHb exposure to mimic the hemorrhage associated microenvironment.

**Figure 6:**
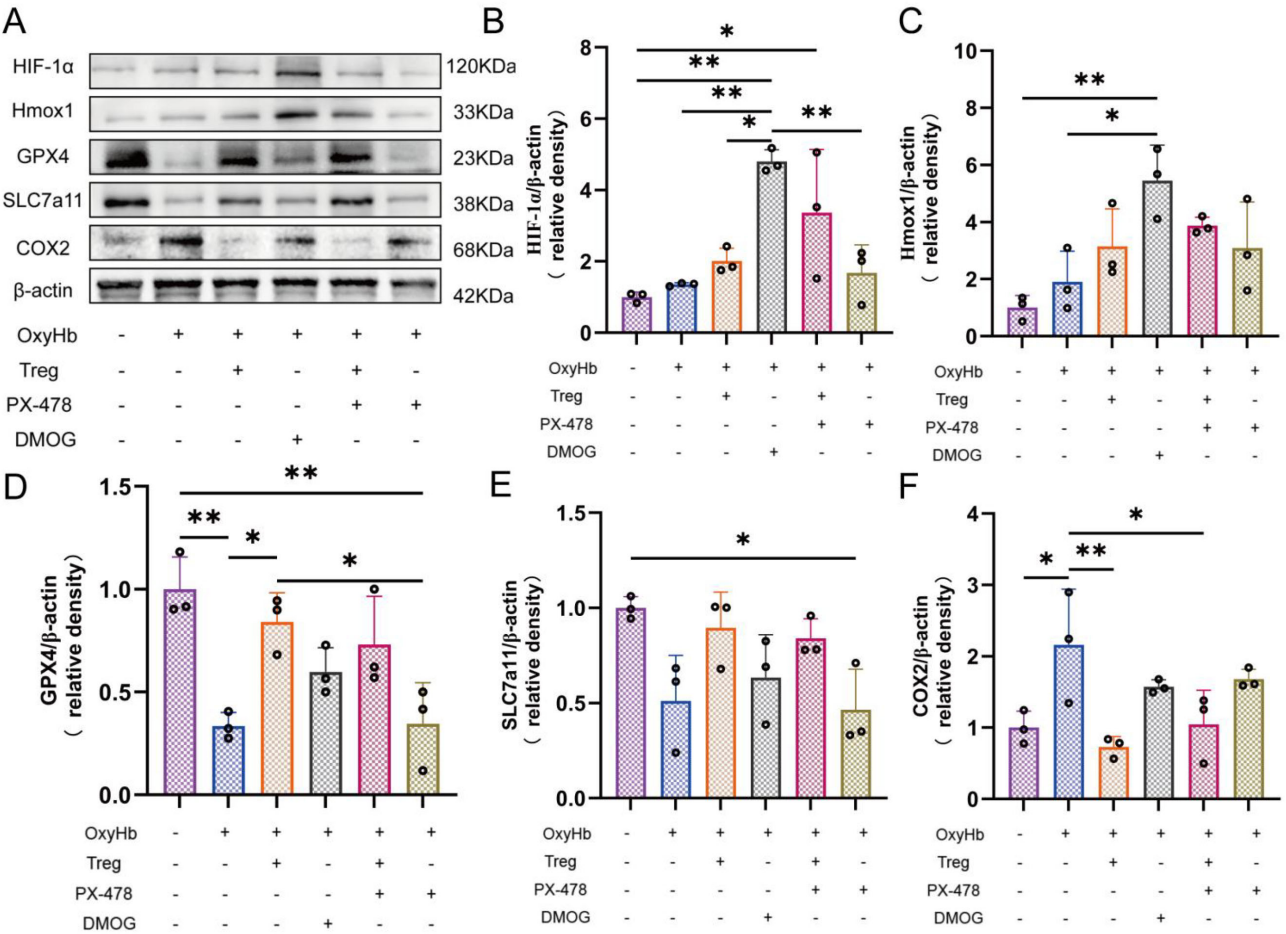
Pharmacological modulation of HIF-1α alters ferroptosis protein expression in astrocytes. **(A)** Western blot analysis following DMOG or PX-478 treatment. **(B–F)** Quantification of HIF-1α, Hmox1, GPX4, SLC7A11, and COX2 expression, n = 3. *P < 0.05, **P < 0.01.

Western blot analysis showed that OxyHb stimulation alone induced a moderate increase in HIF-1α and Hmox1 expression, accompanied by alterations in ferroptosis proteins. Upon DMOG treatment, HIF-1α protein levels were markedly elevated beyond those induced by OxyHb alone, with a concomitant excessive upregulation of Hmox1. Notably, this excessive activation was associated with a pronounced reduction in GPX4 and SLC7A11 expression, together with a significant increase in COX2 levels, indicating an aggravation of ferroptosis molecular changes (**Figures 6A–F**).

In contrast, treatment with PX-478 significantly suppressed HIF-1α expression under OxyHb stimulation, resulting in a marked decrease in Hmox1 levels. This insufficient HIF-1α activity was also accompanied by reduced GPX4 and SLC7A11 expression and elevated COX2 levels, suggesting that excessive inhibition of HIF-1α likewise exacerbated ferroptosis protein dysregulation (**Figures 6A–F**).

Importantly, when PX-478 was applied in the presence of Tregcells, HIF-1α expression was partially restored to an intermediate level compared with PX-478 treatment alone. This moderated HIF-1α expression was accompanied by a relative stabilization of Hmox1 levels, restoration of GPX4 and SLC7A11 expression, and a reduction in COX2 expression. These changes indicate an attenuation of ferroptosis molecular disturbances under Tregcells intervention (**Figures 6A–F**).

Collectively, these results demonstrate that both excessive activation and excessive suppression of HIF-1α signaling exacerbate ferroptosis responses in astrocytes stimulated by OxyHb. In contrast, Tregcells maintains HIF-1α signaling within a relatively balanced range. Thus, it mitigates ferroptosis molecular alterations.

### 3.6 Genetic modulation of HIF-1α reveals a buffering effect on ferroptosis protein dysregulation that depends on Tregcells

**Figure 7:**
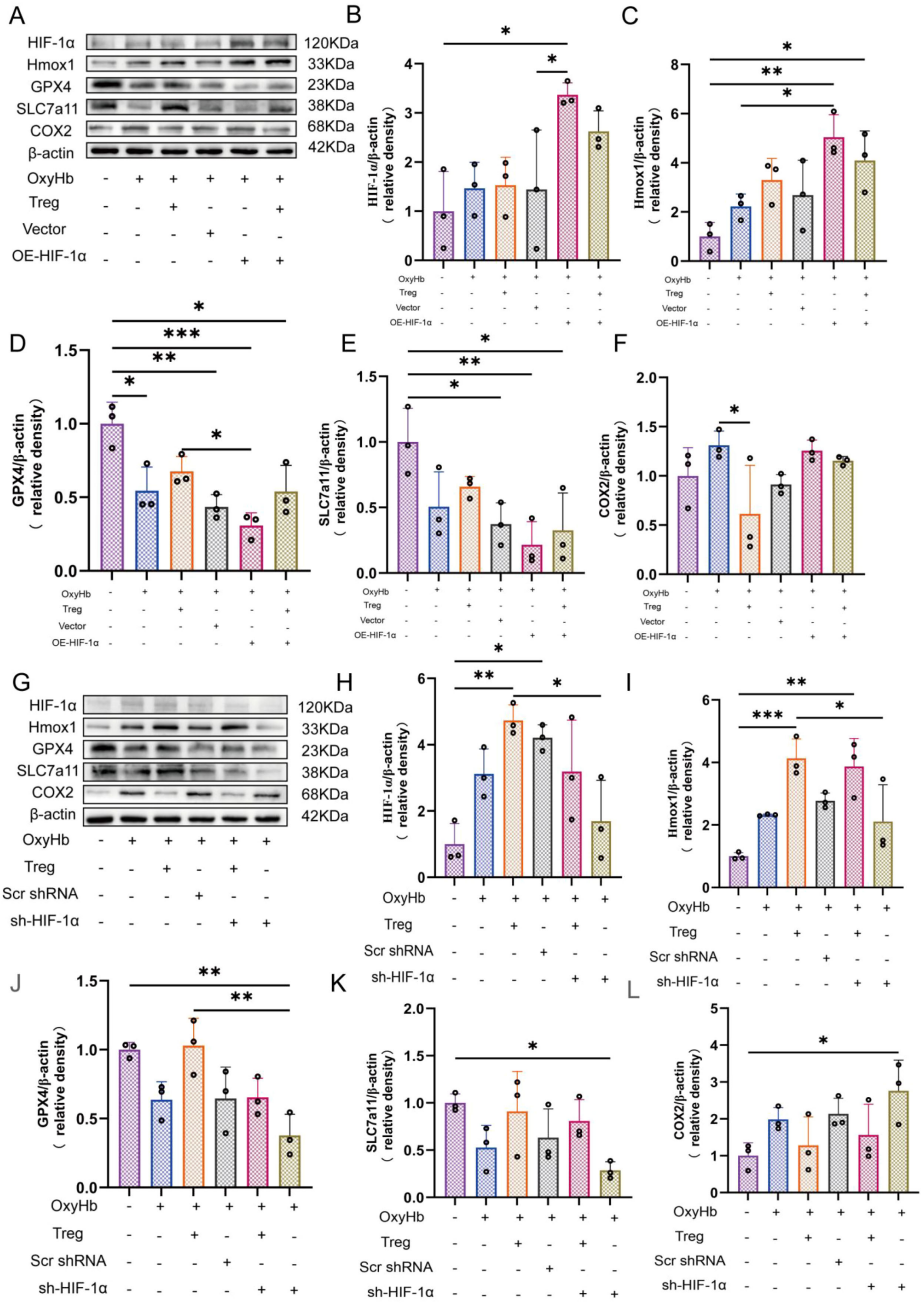
Genetic modulation of HIF-1α alters ferroptosis protein expression in astrocytes. **(A)** Western blot analysis of HIF-1α, Hmox1, GPX4, SLC7A11, and COX2 following HIF-1α overexpression *in vitro*. **(B–F)** Quantification of protein expression normalized to β-actin, n = 3. **(G)** Western blot analysis of ferroptosis proteins following HIF-1α knockdown. **(H–L)** Quantification of protein expression normalized to β-actin, n = 3. *P < 0.05, **P < 0.01, ***P < 0.001.

Astrocytes were transfected with an HIF-1α overexpression plasmid under OxyHb stimulation, and protein expression was assessed by western blotting. OxyHb exposure alone induced moderate alterations in HIF-1α signaling and ferroptosis proteins, whereas Tregcells partially reversed these changes. Astrocytes transfected with empty vector displayed protein expression patterns comparable to those observed in the OxyHb group, indicating no detectable effects from the vector control (**Figure 7A**).

Forced overexpression of HIF-1α resulted in a marked elevation of HIF-1α protein levels, accompanied by a pronounced increase in its downstream target Hmox1. This excessive activation was associated with a significant reduction in the proteins that counteract ferroptosis, GPX4 and SLC7A11, together with a clear upregulation of COX2. These changes indicate aggravated ferroptosis molecular disturbances. Notably, when astrocytes overexpressing HIF-1α were co-cultured with Tregcells, HIF-1α levels were moderately reduced compared with overexpression alone. Meanwhile, Hmox1 expression remained relatively elevated. Under these conditions, GPX4 and SLC7A11 expression were partially restored and COX2 upregulation was attenuated, suggesting that Tregcells mitigates ferroptosis protein dysregulation induced by excessive HIF-1α activation (**Figures 7A–F**).

To assess the effects of insufficient HIF-1α signaling, HIF-1α expression was silenced using shRNA knockdown. As observed previously, the control, OxyHb, and OxyHb plus Tregcells groups exhibited protein expression patterns consistent with earlier experiments, and transfection with scrambled shRNA did not significantly alter HIF-1α or ferroptosis protein levels relative to the OxyHb group (**Figure 7G**).

In contrast, HIF-1α knockdown led to a pronounced reduction in HIF-1α protein levels, accompanied by a marked decrease in Hmox1 expression and dysregulation of ferroptosis proteins, including reduced GPX4 and SLC7A11 expression and increased COX2 levels. Tregcells did not restore HIF-1α expression in HIF-1α deficient astrocytes. However, despite sustained suppression of HIF-1α, the presence of Tregcells was associated with a partial recovery of Hmox1 expression and a concomitant attenuation of ferroptosis protein dysregulation, as evidenced by increased GPX4 and SLC7A11 levels and reduced COX2 expression compared with HIF-1α knockdown alone (**Figures 7G–L**).

Collectively, these genetic manipulation experiments demonstrate that extreme activation or suppression of HIF-1α exacerbates ferroptosis molecular imbalance in astrocytes stimulated with OxyHb. In contrast, Tregcells consistently attenuates these disturbances. This supports a role for Tregcells in stabilizing HIF-1α/Hmox1 signaling and maintaining ferroptosis protein homeostasis under hemorrhagic stress.

### 3.7 Dysregulated Hmox1 expression exacerbates ferroptosis protein alterations in astrocytes stimulated with OxyHb

Hmox1 was identified as a key downstream effector of HIF-1α and one of the most significantly upregulated genes in the transcriptomic analysis. Therefore, we next examined whether direct modulation of Hmox1 expression influences ferroptosis responses in astrocytes. Overexpression and knockdown of Hmox1 using plasmids were performed in astrocytes stimulated with OxyHb, followed by assessment of ferroptosis protein expression by western blotting.

**Figure 8:**
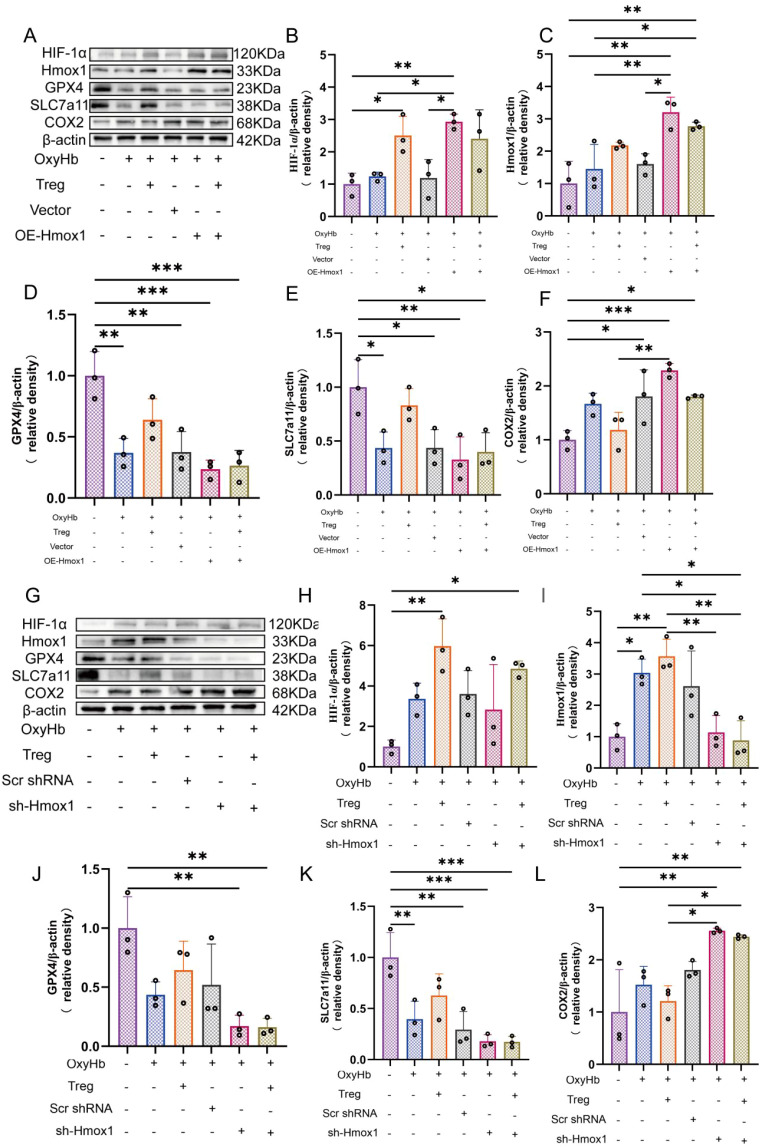
Dysregulated Hmox1 expression aggravates ferroptosis molecular alterations in astrocytes. **(A)** Western blot analysis of HIF-1α, Hmox1, GPX4, SLC7A11, and COX2 following Hmox1 overexpression *in vitro*. **(B–F)** Quantification of protein expression normalized to β-actin, n = 3. **(G)** Western blot analysis of ferroptosis proteins following Hmox1 knockdown. **(H–L)** Quantification of protein expression normalized to β-actin, n = 3. *P < 0.05, **P < 0.01, ***P < 0.001.

In the Hmox1 overexpression experiment, astrocytes exposed to OxyHb displayed moderate alterations in ferroptosis proteins. Tregcells partially normalized these changes. Cells transfected with empty vector exhibited protein expression profiles comparable to those observed in the OxyHb group (**Figure 8A**). Forced overexpression of Hmox1 resulted in a marked increase in Hmox1 protein levels. There was no corresponding change in HIF-1α expression. This excessive induction was accompanied by a significant reduction in GPX4 and SLC7A11 levels and a pronounced upregulation of COX2. This indicated aggravated ferroptosis molecular alterations. Notably, co-culture with Tregcells partially attenuated these changes. This was reflected by a relative preservation of GPX4 and SLC7A11 expression and a reduction in COX2 levels compared with Hmox1 overexpression alone. This occurred despite sustained elevation of Hmox1 (**Figures 8A–F**).

Conversely, shRNA knockdown of Hmox1 led to a substantial reduction in Hmox1 protein expression under OxyHb stimulation. HIF-1α levels remained largely unchanged. This suppression was associated with marked downregulation of GPX4 and SLC7A11 and a significant increase in COX2 expression (**Figures 8G–L**). In contrast to the overexpression condition, Tregcells failed to reverse these ferroptosis protein alterations in astrocytes deficient in Hmox1. Protein expression profiles remained comparable to those observed with Hmox1 knockdown alone. Scrambled control constructs did not induce significant changes relative to the OxyHb group (**Figure 8G**).

Collectively, these findings indicate that both excessive induction and excessive suppression of Hmox1 disrupt ferroptosis protein homeostasis in astrocytes stimulated with OxyHb. Tregcells mitigates ferroptosis molecular disturbances induced by excessive Hmox1 activation. However, it is insufficient to confer protection when Hmox1 expression is markedly reduced. This underscores the requirement for an intact Hmox1 axis in the regulation of ferroptosis mediated by Tregcells.

### 3.8 *In vivo* modulation of Foxp3 alters ferroptosis responses following SAH

**Figure 9:**
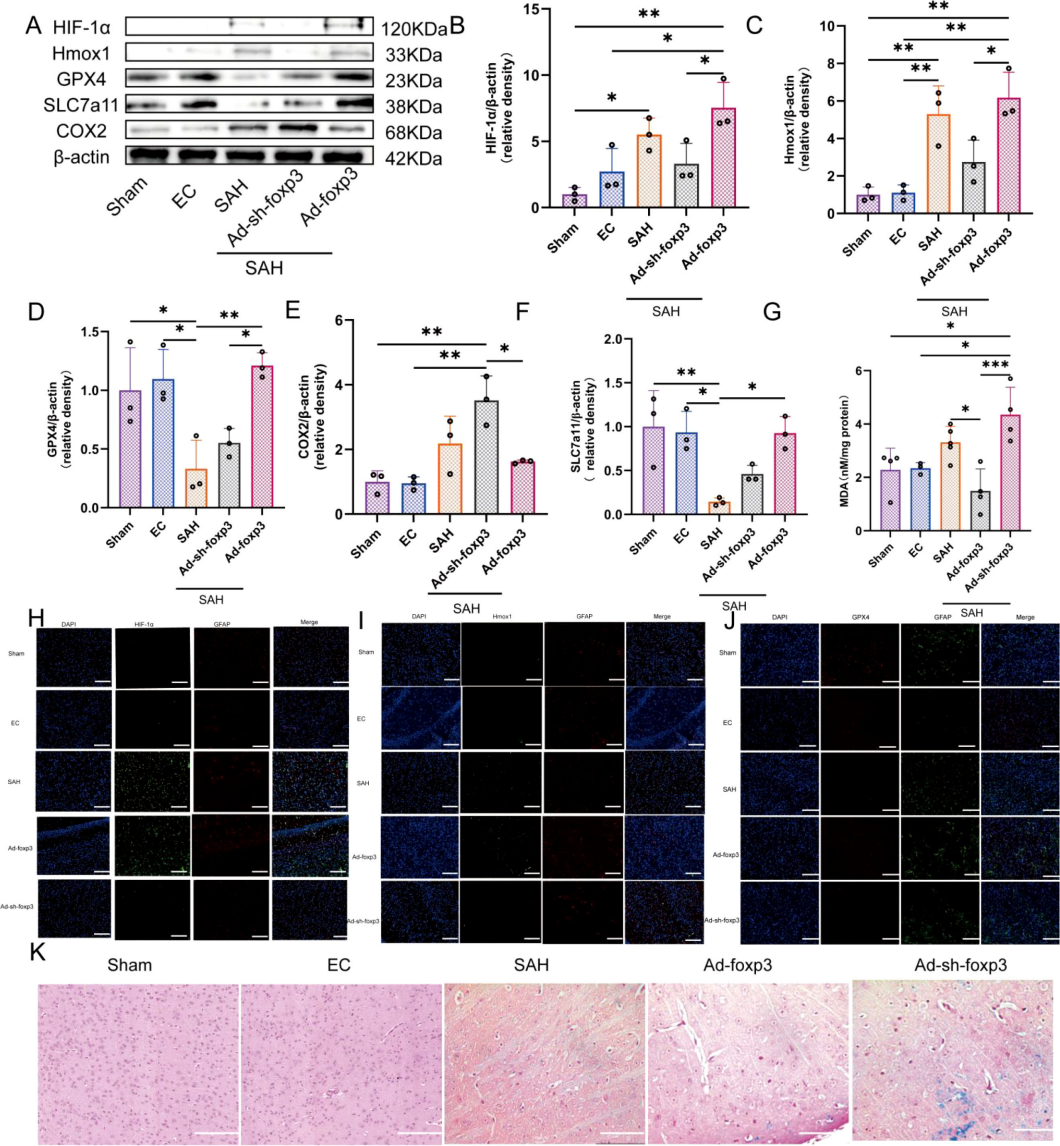
Tregcells regulation dependent on Foxp3 modulates ferroptosis responses after SAH *in vivo*. **(A)** Western blot analysis of HIF-1α, Hmox1, GPX4, SLC7A11, and COX2. **(B–F)** Quantification of protein expression normalized to β-actin, n = 3. **(G)** MDA levels in brain tissue, n =6. **(H–J)** Representative immunofluorescence images showing co-localization with GFAP. **(K)** Representative Prussian blue staining images. *P < 0.05, **P < 0.01, ***P < 0.001.

To determine whether regulation of ferroptosis mediated by Tregcells observed *in vitro* is recapitulated *in vivo*, we selectively manipulated Foxp3 expression using AAV viral vectors. This was done prior to SAH induction. Consistent with the *in vitro* findings, SAH induction alone resulted in moderate alterations in HIF-1α and Hmox1 expression. This was accompanied by downregulation of GPX4 and SLC7A11 and upregulation of COX2 (**Figures 9A–F**). Foxp3 overexpression led to a further elevation of HIF-1α and Hmox1 expression. However, this increase remained within a relatively constrained range compared with the excessive activation observed under direct pharmacological or genetic manipulation *in vitro*. Foxp3 overexpression was associated with restoration of GPX4 and SLC7A11 expression and a reduction in COX2 levels, indicating attenuation of ferroptosis molecular disturbances.In contrast, Foxp3 knockdown showed a trend towards suppression of HIF-1α and Hmox1 expression following SAH. This suppression was accompanied by a modest decrease in GPX4 and SLC7A11 levels. However, these changes did not reach statistical significance. Moreover, COX2 expression further increased in the Ad-sh-Foxp3 group, suggesting exacerbation of ferroptosis molecular dysregulation in the absence of sufficient Tregcells activity (**Figures 9A–F**).

To further assess lipid peroxidation *in vivo*, MDA levels were measured in brain tissue (30). SAH significantly increased MDA content compared with sham controls. Foxp3 overexpression significantly reduced MDA accumulation. In contrast, Foxp3 knockdown led to a further increase in MDA levels, consistent with the observed changes in ferroptosis protein expression (**Figure 9G**).

Immunofluorescence costaining with GFAP was performed to assess astrocytic expression of HIF-1α, Hmox1, and GPX4 *in vivo*. In the sham group, HIF-1α and Hmox1 immunoreactivity was minimal, whereas GPX4 expression was robust in astrocytes. Following SAH, a modest increase in astrocytic HIF-1α was observed. This was accompanied by increased Hmox1 expression and a marked reduction in GPX4 expression. Foxp3 overexpression mediated by AAV resulted in promotion of HIF-1α expression with enhanced Hmox1 levels. This also led to a concomitant restoration of GPX4 immunoreactivity in astrocytes. In contrast, Foxp3 knockdown led to reduced expression of both HIF-1α and Hmox1, together with further suppression of GPX4 (**Figures 9H–J**).

Finally, Prussian blue staining revealed marked iron deposition in brain tissue following SAH. Foxp3 overexpression significantly reduced iron accumulation in the area around the hemorrhage. Foxp3 knockdown aggravated iron deposition, providing histological evidence supporting the biochemical indicators of ferroptosis (**Figure 9K**).

**Figure 10:**
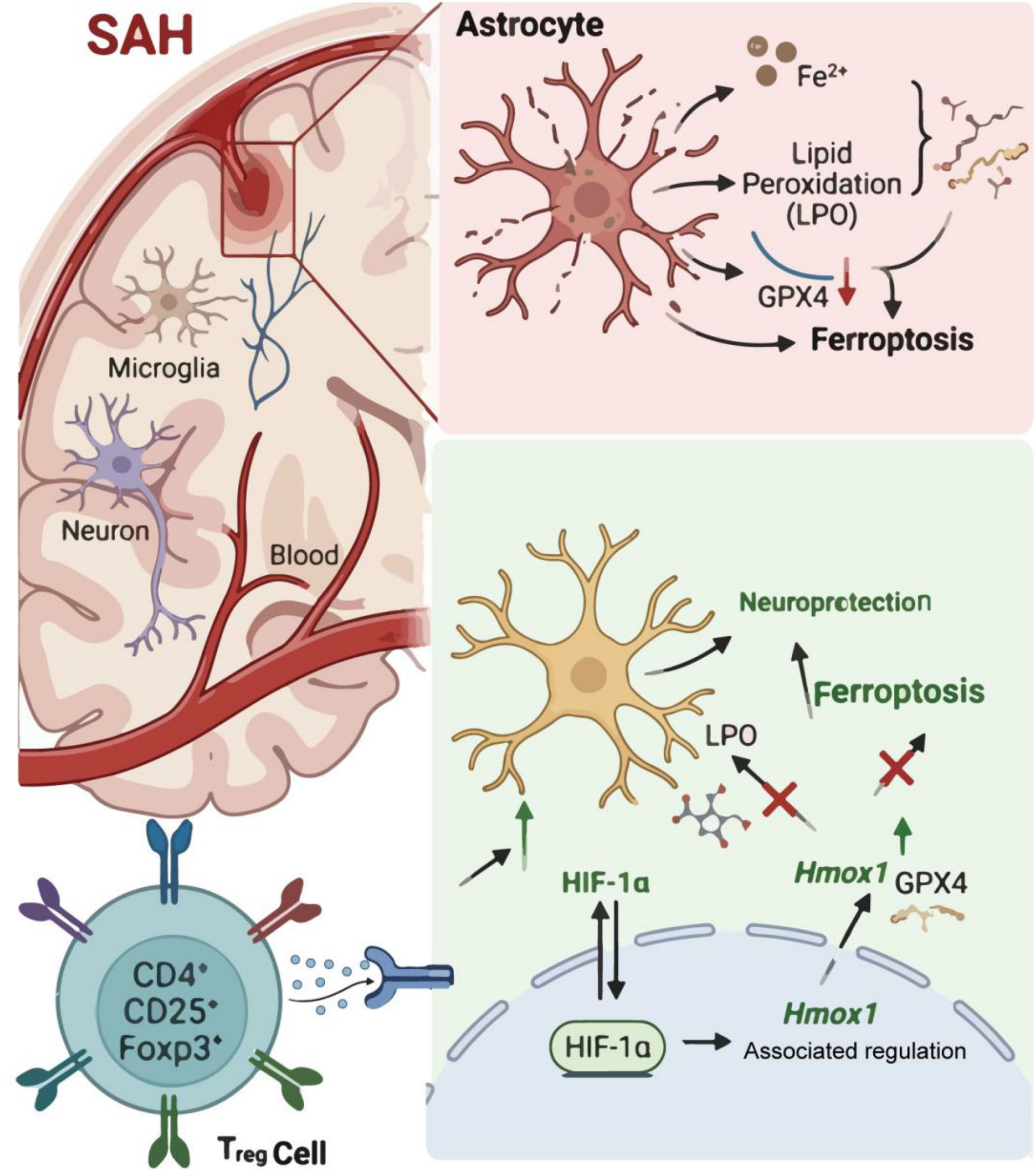
Schematic illustration. Tregcells modulate astrocyte ferroptosis following SAH by stabilizing HIF-1α signaling and enhancing Hmox1 expression, thereby preserving antioxidant defense and limiting lipid peroxidation.

Collectively, these *in vivo* results demonstrate that Tregcells activity dependent on Foxp3 modulates ferroptosis molecular and histopathological changes following SAH. By maintaining HIF-1α/Hmox1 signaling within a controlled range, Tregcells attenuate lipid peroxidation and iron accumulation, thereby limiting ferroptosis brain injury (**Figure 10**).

## 4 Discussion

SAH remains a devastating form of stroke with high mortality and long-term neurological disability. Despite advances in aneurysm management and critical care, effective therapies targeting brain injury after SAH are still lacking (31). Increasing evidence suggests that secondary injury mechanisms, rather than the initial hemorrhagic insult alone, critically determine neurological outcomes. Among these mechanisms, immune dysregulation, oxidative stress, and nonapoptotic cell death pathways have emerged as key contributors. However, how immune responses are coordinated to influence glial survival and redox homeostasis after SAH remains incompletely understood (32). In the present study, we approached this question by integrating *in vivo* immune profiling, *in vitro* modeling focused on astrocytes, transcriptomic analysis, and targeted molecular manipulation. This approach allowing us to delineate a stepwise regulatory axis linking Tregcells to ferroptosis modulation after SAH.

Tregcells have traditionally been viewed as immunosuppressive cells that limit excessive inflammation (33). In the context of SAH, accumulating studies have reported an early increase in Tregcells populations both in the peripheral circulation and within the central nervous system. This increase is generally associated with improved neurological outcomes (34, 35). Emerging evidence indicates that the function of Tregcells after brain injury extends beyond simple suppression of proinflammatory immune responses. Instead, Tregcells appear to actively participate in tissue protection and repair by shaping the local microenvironment (17, 36). Our findings are consistent with this broader view. They demonstrating that Tregcells infiltrate the brain early after SAH and exert a protective influence that cannot be fully explained by inflammation control alone. Our initial experiments established that Tregcells rapidly infiltrate the brain following SAH. Flow cytometry revealed a significant increase in CD4^+^CD25^+^Foxp3^+^ Tregcells as early as 24 h after SAH, with elevated levels persisting through days 5 and 7. This temporal pattern was corroborated by increased Foxp3 protein expression detected by western blotting and by immunofluorescence localization in regions around the hemorrhage. However, we did not perform double staining with cell-type markers. Therefore, we cannot be certain whether all Foxp3 signals came from infiltrating Tregcells. This question will require further investigation.These observations are consistent with previous reports describing early Tregcells recruitment after hemorrhagic stroke, but, they provided the experimental foundation for interrogating Tregcells function beyond their presence alone.

Astrocytes are central regulators of brain homeostasis and are particularly vulnerable to stress induced by hemorrhage. Following SAH, astrocytes are exposed to hemoglobin degradation products, iron overload, and severe oxidative stress, which can profoundly alter their metabolic state and survival (37, 38). Recent studies have highlighted that astrocyte dysfunction after SAH contributes to blood-brain barrier disruption, impaired neuronal support. It also contributes to sustained neuroinflammation (8, 39, 40). Our data suggest that Tregcells directly modulate astrocytic stress responses. This indicates a functional crosstalk between immune cells and glia that shapes astrocyte fate after hemorrhagic injury. To explore how Tregcells influence astrocyte biology under stress related to hemorrhage, we employed an *in vitro* model based on OxyHb. This model recapitulates key aspects of the microenvironment after SAH. Transcriptomic profiling of astrocytes exposed to OxyHb, with or without Tregcells, revealed extensive gene expression remodeling. Notably, GO and KEGG pathway analyses consistently highlighted redox regulation, signaling responsive to hypoxia, and ferroptosis pathways. Among these, the ferroptosis pathway showed the highest enrichment significance, and Hmox1 emerged as one of the most prominently upregulated genes. These unbiased findings driven by data directed our subsequent mechanistic investigations.

One notable finding of this study is the involvement of ferroptosis, a form of regulated cell death dependent on iron and characterized by lipid peroxidation and antioxidant failure. Ferroptosis has increasingly been implicated in hemorrhagic brain injury, where iron release and oxidative imbalance are prominent pathological features (41). Our transcriptomic and biochemical analyses indicated that astrocytes exposed to stress related to hemorrhage exhibit a molecular profile prone to ferroptosis. Tregcells intervention partially reverses these changes. These observations support the notion that inhibition of ferroptosis represents an important component of neuroprotection mediated by Tregcells after SAH. Biochemical validation confirmed that OxyHb exposure induced a molecular signature prone to ferroptosis in astrocytes, characterized by reduced GPX4 and SLC7A11 expression and increased COX2 levels. Tregcells partially reversed these changes, indicating that they modulate astrocytic susceptibility to ferroptosis.This effect was not absolute but rather corrective, suggesting a regulatory role rather than a simple on and off switch for ferroptosis pathways.

At the molecular level, our study highlights the HIF-1α/Hmox1 axis as a critical regulatory node linking immune modulation to ferroptosis control. Both HIF-1α and Hmox1 are molecules responsive to stress with well recognized dual roles. On the one hand, their activation can promote cellular adaptation to hypoxia and oxidative stress; on the other hand, excessive or uncontrolled activation may exacerbate iron release and lipid peroxidation, thereby promoting ferroptosis (37, 42, 43). Our pharmacological and genetic experiments demonstrate that either excessive activation or excessive suppression of HIF-1α or Hmox1 aggravates ferroptosis injury. Tregcells do not exert a uniform suppressive or amplifying effect on the HIF-1α/Hmox1 axis. Rather, regulation mediated by Tregcells is characterized by the maintenance of a relatively stable level of HIF-1α. This is accompanied by a preferential, yet controlled, elevation of Hmox1 expression. This pattern contrasts with the excessive induction observed under direct pharmacological or genetic manipulation. It is associated with preservation of GPX4 expression and attenuation of ferroptosis changes. These findings suggest that Tregcells bias this inherently double edged pathway toward a protective configuration. They do not drive HIF-1α or Hmox1 to levels that promote lipid peroxidation dependent on iron.

The HIF-1α/Hmox1 axis has emerged as a central node linking Tregcells intervention to ferroptosis regulation. Pharmacological manipulation experiments revealed that both excessive activation of HIF-1α (via DMOG) and excessive inhibition (via PX-478) exacerbated ferroptosis protein dysregulation under OxyHb stimulation. Notably, pharmacological inhibition of HIF-1α with PX-478 alone resulted in marked exacerbation of ferroptosis molecular alterations under OxyHb stimulation. However, when PX-478 was applied in the presence of Tregcells, this phenotype that promotes ferroptosis was partially reversed. Tregcells intervention mitigated the excessive suppression of HIF-1α signaling and restored downstream antioxidant capacity. This indicates that they can counterbalance pharmacological perturbation. They also preserve a functionally favorable level of pathway activity rather than requiring complete pathway integrity. These findings were independently corroborated by genetic manipulation experiments, in which both overexpression and knockdown of HIF-1α resulted in reduced GPX4 and SLC7A11 expression and elevated COX2 levels. Together, these data indicate that HIF-1α exerts a bidirectional, dose sensitive influence on ferroptosis rather than a linear protective effect (44).

Downstream of HIF-1α, direct manipulation of Hmox1 yielded a similar pattern. Both enforced overexpression and silencing of Hmox1 aggravated ferroptosis protein alterations in astrocytes stimulated with OxyHb. This occurred despite leaving HIF-1α levels largely unchanged. This finding is particularly relevant given the dual biological role of Hmox1 in antioxidant defense and iron release. It supports the concept that inappropriate Hmox1 expression, whether excessive or insufficient, can destabilize redox homeostasis and promote ferroptotic injury.

Crucially, these mechanistic insights were recapitulated *in vivo* through modulation of Tregcells activity mediated by Foxp3. AAV-driven Foxp3 overexpression resulted in increased astrocytic HIF-1α induction, enhanced Hmox1 expression, and restoration of GPX4 levels after SAH. In contrast, Foxp3 knockdown led to coordinated suppression of HIF-1α and Hmox1 and further loss of GPX4. These molecular changes were accompanied by corresponding alterations in lipid peroxidation and iron deposition, as evidenced by MDA measurements and Prussian blue staining. Importantly, the *in vivo* data mirrored the *in vitro* observation that protection was associated with balanced, rather than maximal, activation of the HIF-1α/Hmox1 axis.One limitation regarding our ferroptosis assessment is that we did not directly measure free Fe²^+^ levels (45). Although Prussian blue staining detects iron deposition. However, it does not reflect the labile ferrous iron pool that directly drives lipid peroxidation. Future studies incorporating ferrous iron-specific probes or assays would provide a more complete picture of iron dysregulation in this model.

Taken together, our findings support a model in which Tregcells act as fine tuners of astrocytic stress responses after SAH. By restraining the extremes of HIF-1α/Hmox1 signaling, Tregcells shift these inherently double edged pathways toward a protective state. They thereby limiting ferroptosis and mitigating secondary brain injury. This work expands the current understanding of Tregcells function in SAH, emphasizing their role as active regulators of glial metabolism and cell death rather than passive suppressors of inflammation. Targeting interactions between Tregcells and astrocytes or restoring balanced HIF-1α/Hmox1 signaling may therefore represent a promising therapeutic strategy for SAH.

This study identifies Tregcells as active modulators of astrocytic ferroptosis following SAH. Tregcells limit ferroptosis by stabilizing HIF-1α signaling while selectively enhancing Hmox1 expression, thereby preserving antioxidant capacity dependent on GPX4. Excessive activation or suppression of this pathway exacerbates ferroptosis under hemorrhagic stress. This is a novel insight which may provide new approaches for the treatment of SAH.Our findings suggest two translational possibilities: enhancing Tregcells function or fine-tuning the HIF-1α/Hmox1 axis. Before clinical application, we need to test whether the protective effect works with clinically relevant Tregcells products, determine the optimal timing and dose, and assess safety in the phase of SAH.

## 5 Limitations

There are some limitations to this study. First, we did not include classical ferroptosis inducers as positive controls, although our multi-level data support ferroptosis involvement. Second, the association between astrocytic ferroptosis and neurological outcomes is correlative rather than causal. Third, the precise molecular mechanisms governing Tregcells-astrocyte crosstalk remain unclear. Our transcriptomic data may provide clues for future investigation, but dedicated mechanistic studies are needed.

## Data Availability

The raw data supporting the conclusions of this article will be made available by the authors, without undue reservation.
