# Supplementary figures and images for: Treg cells protect astrocytes from ferroptosis after subarachnoid hemorrhage by activating the HIF-1α/Hmox1 pathway
Source: Front Immunol. 2026 May 22;17:1825459. doi: 10.3389/fimmu.2026.1825459 (PMC13236614; doi:10.3389/fimmu.2026.1825459)

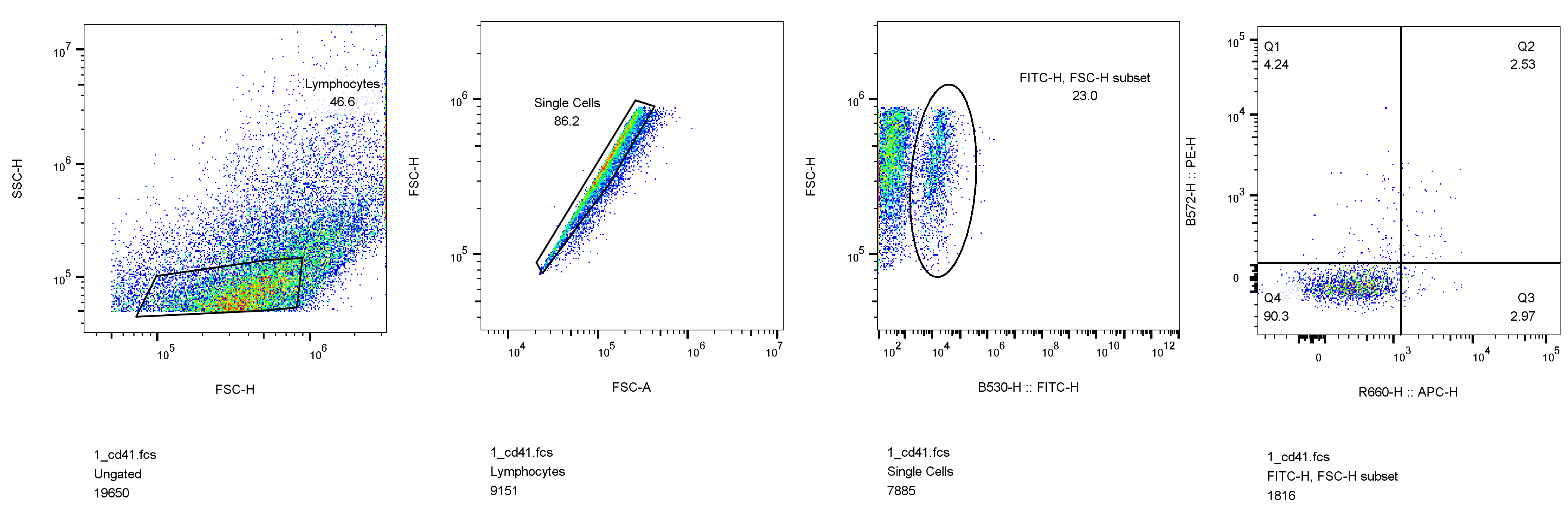

Supplement: Supplementary file 1 [file DataSheet1.zip › Supplementary/Figure 1.tiff]
